# Mechanosensitive Ion Channels: Molecular Hubs Integrating Skeletal Muscle Adaptation and Systemic Homeostasis

**DOI:** 10.7150/ijbs.127875

**Published:** 2026-04-08

**Authors:** Chong Zhao, Jinying Zhang, Xu Wang, Hongzhen Li, Jie Huang, Jiacheng Wang, Jianhao Wang, Haoran Zhang, Yan Zeng, Haiying Liu, Shuai Xu

**Affiliations:** 1 Department of Spinal Surgery, Peking University People's Hospital, Peking University, Beijing, PR China.; 2 Beijing Key Laboratory for Surgical Navigation Robots with Augmented Reality, School of Optics and Photonics, Beijing Institute of Technology, 100081 Beijing, China.; 3 Yangtze Delta Region Academy of Beijing Institute of Technology, 314001 Jiaxing, Zhejiang, PR China.; 4 School of Pharmacy, Hebei Medical University, Shijiazhuang, PR China.

**Keywords:** mechanotransduction, Piezo, TRP, skeletal muscle, myokines, calcium signaling, muscle regeneration

## Abstract

Skeletal muscle's ability to perceive and adapt to physical force is fundamental to tissue homeostasis and systemic health. At the core of this process, mechanosensitive ion channels (MSCs)—notably the Piezo and TRP families—function as primary transducers. This review synthesizes how these channels convert diverse mechanical stimuli into biochemical signals. We delineate how their activation, primarily through Ca2+ influx, engages downstream signaling hubs, including the Hippo-YAP/TAZ, MAPK, and PI3K-Akt-mTOR pathways. These cascades subsequently orchestrate muscle growth, regeneration, and metabolic remodeling.

We then bridge these molecular mechanisms to clinical relevance, analyzing how physical therapies like low-intensity pulsed ultrasound and electrical stimulation precisely target these networks to enhance muscle repair. Furthermore, we explore the role of MSCs in driving skeletal muscle's function as an endocrine organ. Mechanical activation triggers myokine release, mediating critical inter-organ communication with bone, adipose, and immune systems. Collectively, this review establishes MSCs as pivotal molecular hubs that integrate external physical energy with local tissue repair and systemic physiological regulation.

## Introduction

Living cells are able to detect and respond to mechanical cues, a process termed mechanosensation, which is central to development, tissue repair, and regeneration^1^. While virtually all cell types utilize mechanosensitive ion channels (MSCs) for basic homeostatic functions, such as cell volume regulation and tactile sensing, the role of these channels is exceptionally specialized in skeletal muscle. Unlike most static tissues, skeletal muscle is a highly dynamic organ subjected to constant, dramatic, and variable physiological loading—ranging from forceful macroscopic contractions and extreme stretch to external compression and shear stress 1,2. In this uniquely demanding mechanical environment, external forces are translated into intracellular biochemical signals, enabling cells to adjust gene expression and functional states according to their dynamic surroundings. Mechanical inputs such as extracellular matrix (ECM) elasticity and stretching are especially important in skeletal muscle, where they regulate the activity of muscle stem cells and thereby influence growth, repair, and functional maintenance 3. This conversion of physical forces into intracellular signaling is known as mechanotransduction. A key step in this process is mediated by mechanosensitive ion channels (MSCs), specialized transmembrane proteins positioned at the cell-environment interface 4. Acting as direct molecular sensors, MSCs respond to stimuli such as membrane stretch, tension, and osmotic stress by undergoing conformational changes that open the channel pore, allowing ion flux across the membrane 4. This rapid ion movement converts a mechanical input into an electrochemical signal, initiating downstream pathways that coordinate cellular adaptation 4.

Multiple MSC families have been identified, including Piezo, transient receptor potential (TRP), two-pore domain potassium (K2P), and the more recently discovered Transmembrane Protein 63 (TMEM63, or OSCA in plants) channels 5. These channels form a diverse signaling network that enables skeletal muscle to sense and respond to a broad spectrum of mechanical cues. Activation of mechanosensitive channels by physiological loading or therapeutic interventions engages complex downstream networks that regulate key cellular decisions such as proliferation, differentiation, and survival, processes that are fundamental for muscle adaptation and homeostasis 6. Beyond local regulation, skeletal muscle also functions as an endocrine organ: mechanical stimuli promote the secretion of myokines, which act on distant organs—including the immune system—to influence systemic health and inflammation 7.

Recent advances have greatly expanded our understanding of mechanosensitive ion channels in skeletal muscle, revealing their structural and functional diversity, activation by distinct physical cues, and integration into downstream signaling cascades. Moreover, skeletal muscle is increasingly recognized as a secretory organ that mediates inter-organ communication. These perspectives together underscore the central roles of mechanosensitive ion channels in muscle biology and highlight their potential as therapeutic targets, while outlining the key challenges that remain for clinical translation.

## Mechanosensitive Ion Channels in Skeletal Muscle: Classification, Functions, and Synergistic Roles

**Figure 1:**
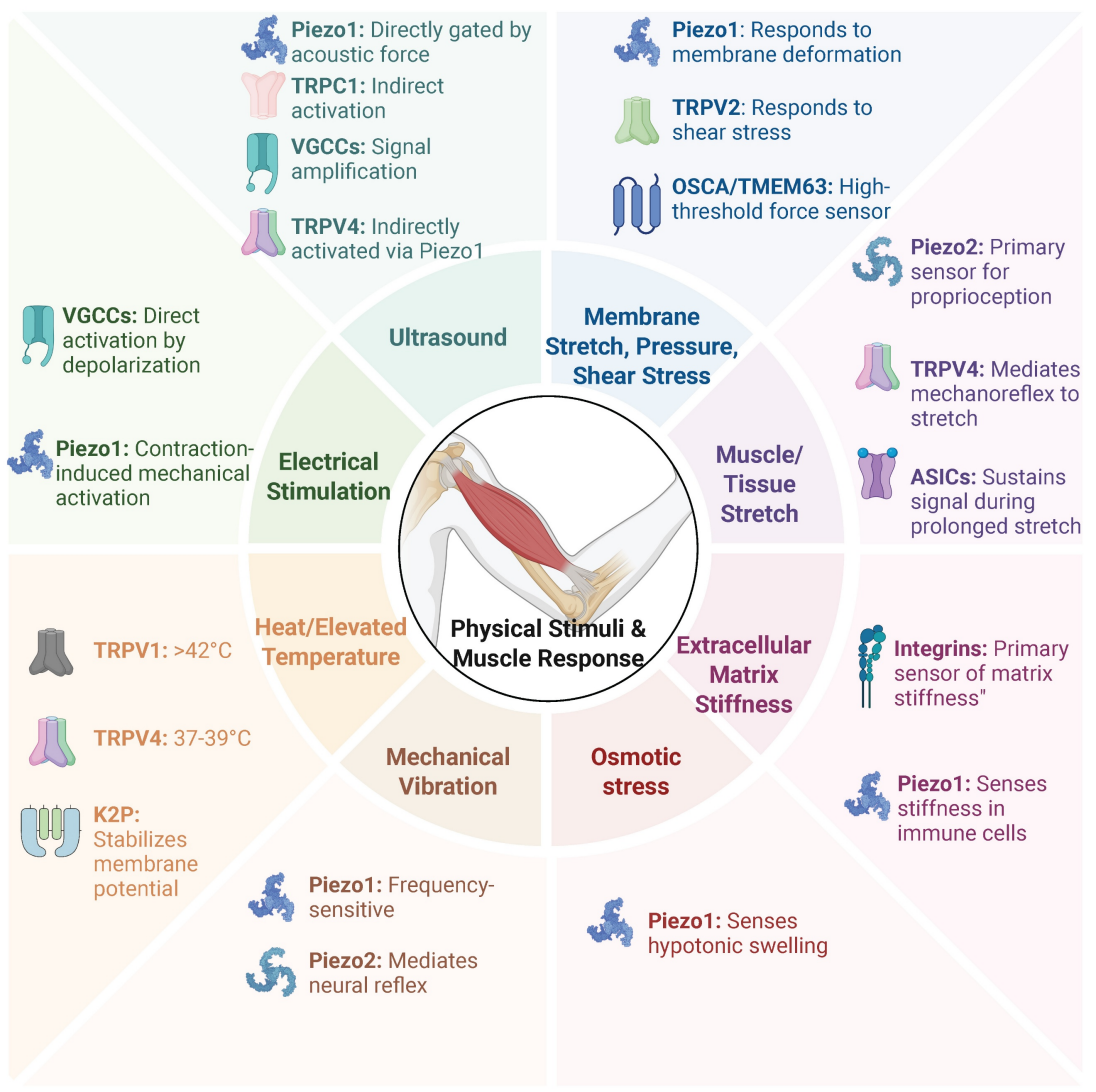
** Primary physical stimuli and their target mechanosensitive receptors in skeletal muscle.** Skeletal muscle utilizes an integrated network of mechanosensitive ion channels (MSCs) and receptors (such as Piezo, TRP, and VGCCs) to decode mechanical cues into adaptive physiological responses. This figure summarizes the primary physical stimuli and their corresponding target receptors.

Multiple families of MSCs, including Piezo, TRP, K2P, and TMEM63/OSCA channels, have been characterized in mammals 4. In skeletal muscle, these channels—together with other mechanosensitive receptors such as Purinergic receptor P2X7 (P2X7) and Voltage-gated calcium channels (VGCCs) - constitute an integrated signaling network that decodes mechanical cues into adaptive physiological responses 8. The primary physical stimuli and the mechanosensitive receptors they target are summarized in Figure 1.

### Gating Mechanisms: "Force-from-Lipid" versus the "Tethered" Model

**Figure 2:**
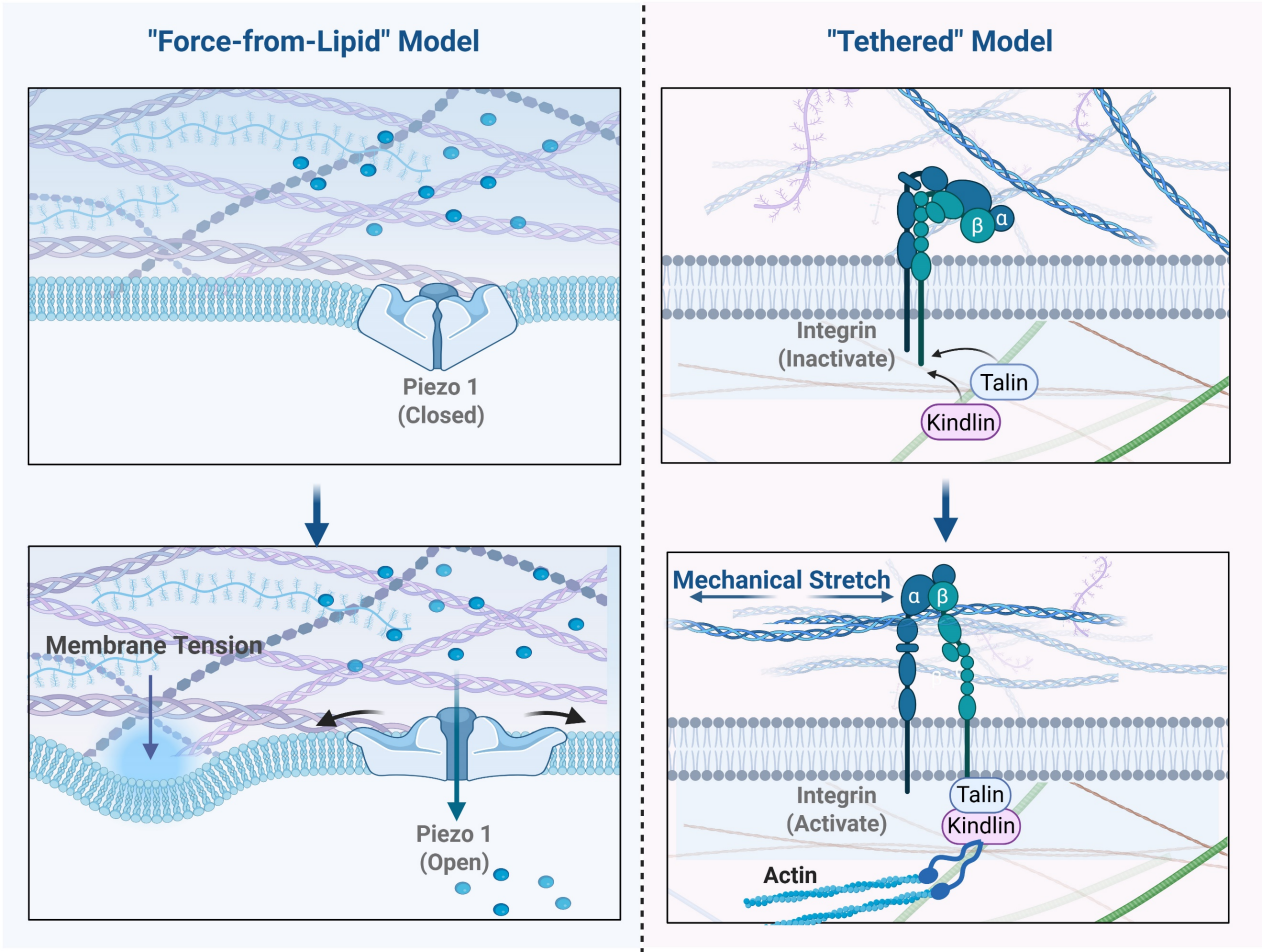
** Gating Mechanisms: "Force-from-Lipid" versus the "Tethered" Model.** This figure contrasts the two primary theories of ion channel mechanogating. (A) "Force-from-Lipid" Model: Exemplified by Piezo1. In the resting state (left), Piezo1's "blades" create a "nanodome" in the membrane. Increased Membrane Tension (right) flattens the membrane, pulling the central pore open via a lever-like action. (B) "Tethered" Model: Exemplified by the integrin-cytoskeleton linkage. In the resting state (left), tethers are slack. Force from Mechanical Stretch (right) is transmitted from the ECM through integrin to the cytoskeleton. The cytoskeleton acts as a taut "tether" to directly pull the ion channel open.

Mechanistically, the activation of ion channels by physical force is understood through two primary models: the "force-from-lipid" model and the "tethered" model. The core distinction lies in how force is transmitted to the channel protein (Figure 2).

The "force-from-lipid" model posits that mechanical forces act directly on the lipid bilayer of the cell membrane. Changes in membrane tension, curvature, or thickness drive conformational changes in the embedded channel protein, leading to its opening. In this process, the lipid bilayer itself is the medium of force transmission, theoretically requiring no participation from other accessory proteins 9.

In contrast, the "tethered" model proposes that force is transmitted via ancillary structures linked to the channel 10. These "tethers" can be extracellular matrix (ECM) proteins or intracellular cytoskeletal components. When the cell is mechanically stretched, these connecting structures act like ropes, directly pulling on specific domains of the channel to induce its opening 9. Classic examples include the transduction channels in auditory and vestibular hair cells, which are linked to neighboring stereocilia by extracellular "tip links" and to the intracellular actin cytoskeleton. Similarly, low-threshold mechanoreceptors (LTMRs) in the skin require protein filaments connecting the channel to the ECM to function 10.

Initially, due to the complex cytoskeletal and ECM networks in eukaryotic cells, the "tethered" model was widely favored. Integrins, as key transmembrane proteins linking the ECM to the cytoskeleton, were considered central hubs in this model, theoretically transmitting external stress through the cytoskeleton to ion channels. Indirect evidence, such as the observation that cytoskeleton-disrupting drugs alter the mechanosensitivity of some channels, seemed to support this view. However, despite its theoretical appeal, direct experimental evidence for the tethered model in mammalian skeletal muscle cells remains limited 11.

This evidentiary gap has shifted focus towards the "force-from-lipid" model, which has gained substantial and compelling experimental support. This model was firmly established by studies of the prokaryotic mechanosensitive channels MscL and MscS in *E. coli*. These channels were shown to be effectively activated by membrane tension even after being purified and reconstituted into artificial liposomes, which completely lack a cytoskeleton or any other protein components. This unequivocally demonstrated that their activation depends solely on the interaction between the channel protein and the surrounding lipid bilayer 12. Importantly, this lipid-channel interaction is highly sensitive to the bilayer's specific physical properties. As demonstrated by studies on MscL and MscS, alterations in membrane thickness and lipid composition—such as changes in cholesterol levels—can significantly alter bilayer stiffness and transbilayer pressure profiles, thereby directly tuning the channel activation thresholds 12. Extending this principle to skeletal muscle, the highly specialized local lipid microenvironments within the cell—most notably the transverse tubules (T-tubules), which possess unique lipid compositions and high cholesterol content—likely play a crucial role in locally modulating the mechanosensitivity of resident ion channels. Crucially, the 'force-from-lipid' principle is not limited to prokaryotes but is evolutionarily conserved in mammalian mechanosensors, as evidenced by high-resolution structural studies. For instance, the mammalian Piezo1 channel forms a unique trimeric propeller that creates a localized 'nanodome' depression in the lipid bilayer. According to the prevailing model, increased membrane tension flattens this dome, exerting a lever-like force on the channel blades to open the central pore 10,13. Similarly, the two-pore domain potassium channels (K2P), such as TREK-1 and TRAAK, utilize a distinct 'lipid-cork' gating mechanism. In the closed state, an acyl chain from the membrane physically plugs the ion pathway; upon membrane stretch, this lipid cork is displaced, allowing ion conduction 14. These structural insights confirm that for both Piezo and K2P families, the lipid bilayer itself is the primary transducer of mechanical force.

In summary, a large body of evidence establishes the "force-from-lipid" model as a fundamental and widespread activation mechanism. This does not entirely invalidate the "tethered" model. A more comprehensive modern view is that the two models are not mutually exclusive but may act in concert. A channel might be primarily activated by the "force-from-lipid" mechanism, while its localization, activation threshold, or integration with other signaling pathways could be fine-tuned by the cytoskeleton or ECM. Understanding these precise activation mechanisms is critical for skeletal muscle, which experiences dramatic mechanical changes. Future research, guided by the "force-from-lipid" model as a core principle while incorporating the modulatory role of the "tethered" model, will provide a more complete framework for understanding mechanotransduction in skeletal muscle and beyond.

### Primary Mechanosensors: The Piezo Protein Family

The Piezo family of proteins represents a pivotal class of mechanotransducers in mammalian cells 15,16. This family is comprised of two main subtypes: Piezo1, which is predominantly expressed in non-sensory tissues exposed to mechanical forces, and Piezo2, which is primarily found in sensory tissues 17. Structurally, these channels function as distinct mechanotransducers that directly sense membrane tension via the "force-from-lipid" mechanism described in Section 2.1, without requiring cytoskeletal tethers^18^. Upon opening, they exhibit a slight preference for Ca^²⁺^ 17,19. The resulting ion flux, particularly of calcium, converts the physical stimulus into an electrochemical signal that initiates downstream signaling pathways 18,20.

Piezo1 plays multiple, critical roles in skeletal muscle, spanning myogenesis, regeneration, and the regulation of muscle mass 21,22. Its function is evident in both the muscle stem cell (MuSC) niche and mature myofibers. In quiescent MuSCs, Piezo1 activity is indispensable for maintaining the stem cell pool; its deletion markedly reduces stem cell numbers and elevates reactive oxygen species (ROS), leading to DNA damage and p53-dependent senescence 22. Piezo1 is expressed in quiescent MuSCs, and its deletion markedly reduces stem cell numbers, highlighting its essential role in self-renewal and maintenance. Furthermore, Piezo1-mediated Ca²⁺ influx enables the assembly of the actomyosin network via the RhoA/ROCK signaling pathway, a necessary step for myoblast fusion and myotube elongation 22. Beyond development, Piezo1 acts as a cellular "mechanostat" in mature myofibers to balance muscle maintenance and atrophy. Mechanical unloading suppresses Piezo1 expression, lowering basal intracellular Ca²⁺ levels and upregulating the transcription factor KLF15, which subsequently activates IL-6 transcription to promote muscle atrophy 21. This mechanism is supported by pharmacological evidence where the inhibitor GsMTx-4 mimics the atrophic phenotype, while Yoda1 blunts the upregulation of atrophic genes. Crucially, human biopsy data from limb-casting patients confirm these findings, showing reduced PIEZO1 mRNA and increased atrogene expression 21. Collectively, this evidence suggests that Piezo1 acts as a key sensor in mature myofibers that translates mechanical load into signals that suppress atrophic pathways and maintain muscle mass.

While Piezo1 governs tissue remodeling, Piezo2 is recognized as the principal mechanotransduction channel responsible for proprioception 23. It functions as the primary sensor within muscle spindles, converting muscle stretch into nerve impulses that inform the central nervous system about body position and movement 23. Loss-of-function mutations in human PIEZO2 lead to severe deficits, including muscular atrophy and scoliosis 23. Although Piezo2 is the primary sensor, its rapid adaptation suggests that it functions in concert with other molecular elements, such as ASICs, to maintain sustained firing during prolonged stretch, ensuring accurate motor control 23.

### Multimodal Sensors of Physical Stimuli: The TRP Channel Family

The Transient Receptor Potential (TRP) superfamily consists of non-voltage-gated cation channels that act as multimodal sensors for a wide array of physical and chemical stimuli 24. In skeletal muscle, the vanilloid subfamily (TRPV)—particularly TRPV1, TRPV2, and TRPV4—are the most consistently detected and functionally significant isoforms.

TRPV1, best known as the capsaicin receptor, functions as a polymodal sensor activated by heat, acidosis, and chemical ligands 24-26. In skeletal muscle, it localizes to the sarcoplasmic reticulum (SR) membrane, functioning as a Ca²⁺-leak channel that engages in crosstalk with ryanodine receptor 1 (RyR1) to regulate intracellular Ca²⁺ release 26. Functionally, TRPV1 activation is directly linked to metabolic adaptation. For instance, agonists like eugenol initiate a calcineurin (CaN)-mediated signaling pathway that promotes fast-to-slow muscle fiber remodeling and enhances IL-15 expression, mimicking the effects of exercise. Similarly, capsaicin activates TRPV1 to elevate the expression of Uncoupling Protein (UCP) and ATP-dependent thermogenic proteins (e.g., SERCA, RyR), thereby enhancing non-shivering thermogenesis 27. Furthermore, TRPV1 upregulation during cold stress acclimation underscores its critical role in thermal and metabolic adaptation 28.

In contrast to the metabolic role of TRPV1, TRPV2 acts as a critical mechanosensor during the early stages of myogenesis 29. Its expression peaks in proliferating myoblasts and declines upon differentiation. Mechanistically, TRPV2 responds to mechanical cues—such as fluid flow-induced shear stress—by mediating a rapid increase in cytosolic Ca²⁺ 29. This mechanosensitive Ca²⁺ influx serves as a prerequisite integrator of mechanical signals, essential for the subsequent fusion of myoblasts into mature myotubes during the initial phases of muscle formation.

Finally, TRPV4 functions as a dual sensor of mechanical and thermal stimuli within the muscle microenvironment. It is highly expressed in dorsal root ganglion (DRG) neurons innervating skeletal muscle, where it mediates the muscle mechanoreflex. Pharmacological inhibition of TRPV4 markedly attenuates afferent discharge and blunts the sympathetic response to passive stretch, indicating that TRPV4 helps sense mechanical distortion in working muscle 30. Additionally, TRPV4 acts as a thermosensor in the vasculature supplying skeletal muscle. In isolated human feed arteries, physiological warming from 37 °C to 39 °C inhibits α1-adrenergic vasoconstriction—a phenomenon termed "heat-induced sympatholysis." This vasodilatory response is abolished by specific TRPV4 inhibition or endothelial denudation, demonstrating that endothelial TRPV4 channels are responsible for optimizing blood flow during heat stress 31.

### Integrins: Structural Links for Mechanotransduction

Integrins function as non-channel mechanotransducers that physically link the extracellular matrix (ECM) to the intracellular actin cytoskeleton 32. This connection is primarily organized at specialized sites called focal adhesions (FAs), which serve as critical hubs for bidirectional signaling and force transmission 33. In skeletal muscle, β1-containing integrins are concentrated at specialized force-transducing hubs, such as costameres and myotendinous junctions (MTJs) 34.

Unlike ion channels, integrins operate through a structural gating mechanism, switching between a bent, low-affinity conformation and an extended, high-affinity state competent for ligand binding 35. This activation is bidirectional: it can be triggered by "outside-in" signals (ECM binding) or "inside-out" signals, where intracellular co-activators like talin and kindlins bind the β-subunit tail to induce the active conformation 32,34,35. Through this mechanism, integrins translate mechanical tension into biochemical outputs.

Upon activation, the clustering of integrins initiates the assembly of the "integrin adhesome," a dynamic complex centered on Focal Adhesion Kinase (FAK) 36. FAK autophosphorylation triggers a cascade that engages canonical signaling modules, including the MAPK/ERK and PI3K-Akt-mTOR axes, to regulate cell survival and differentiation 34,36. Crucially, the integrin-actin axis serves as a mechanical checkpoint for the Hippo pathway; cytoskeletal tension transmitted through integrins dictates the nuclear translocation of YAP/TAZ. This coupling ensures that transcriptional programs are strictly aligned with the physical state of the muscle niche 33,37.

Furthermore, integrin signaling is functionally integrated with mechanosensitive ion channels. For instance, Ca²⁺ influx through activated Piezo1 channels activates the protease calpain, which subsequently cleaves talin. This cleavage uncouples integrins from the cytoskeleton, leading to a localized downregulation of adhesion 38. This crosstalk demonstrates a sophisticated feedback loop where ion channels and structural receptors coordinate to fine-tune the cellular response to mechanical loading.

### Voltage-Gated Calcium Channels: Essential Amplifiers of Mechanical Signals

VGCCs are multi-subunit transmembrane proteins crucial for converting electrical signals into intracellular Ca²⁺ transients 39,40. While not primary mechanosensors that gate directly in response to membrane stretch, VGCCs—specifically the Cav1 (L-type), Cav2 (P/Q-, N-, and R-type), and Cav3 (T-type) subfamilies—function as indispensable secondary transducers. They amplify mechanical signals initiated by upstream sensors, serving as a critical link between initial mechanosensation and robust downstream cellular responses 39,41,42.

This amplification role is best exemplified by the response to physical stimuli like pulsed focused ultrasound (pFUS). Mechanistically, pFUS does not directly gate VGCCs; instead, it activates the mechanosensitive TRPC1 channel, generating a localized Na⁺-driven depolarization. This electrical shift subsequently gates adjacent L-type Ca²⁺ channels, triggering a massive Ca²⁺ influx necessary for downstream effects such as COX2 upregulation and mesenchymal stromal cell tropism 43. Experimental evidence confirms that this response is abolished by VGCC blockers, extracellular Na⁺ depletion, or TRPC1 suppression, defining a specific "TRPC1-VGCC axis" where VGCCs convert a localized mechanosensitive current into a widespread Ca²⁺ signal 43,44.

Beyond acute signaling, VGCCs drive long-term developmental remodeling at the neuromuscular junction (NMJ). During postnatal synapse elimination, L-type (CaV1.3) and P/Q-type (CaV2.1) channels mediate the activity-dependent pruning of redundant axons. Pharmacological blockade of these channels markedly delays nerve terminal retraction, while their activation accelerates it, identifying Ca²⁺ influx as the key regulator of synaptic refinement. Furthermore, specific regulatory subunits, such as the embryonic CaVβ1, are required for acetylcholine receptor (AChR) pre-patterning, ensuring the structural stability of the mature NMJ 40,41,45.

### Mechanotransduction and Mitochondrial Homeostasis in Pathology

Beyond cytosolic Ca²⁺ signaling, the functional coupling between cell surface mechanosensors and mitochondria is emerging as a critical determinant of tissue homeostasis. Mechanically induced Ca²⁺ influx via MSCs (e.g., Piezo1, VGCCs) can be rapidly taken up by mitochondria through the mitochondrial calcium uniporter (MCU). While physiologic Ca²⁺ transients stimulate bioenergetics, aberrant mechanosensing leads to mitochondrial dysfunction, a key driver in diverse pathologies ranging from myopathies to vascular diseases 46,47.

Recent evidence highlights that the disruption of this axis contributes to specific disease phenotypes through mechanisms involving oxidative stress and regulated cell death. For instance, in models of doxorubicin-mediated injury, the overactivation of Piezo-type mechanosensitive channels triggers mitochondrial Ca²⁺ overload. This breakdown of mitochondrial quality surveillance leads to the opening of the mitochondrial permeability transition pore (mPTP) and subsequent necroptosis or ferroptosis, processes that can be mitigated by specific targeting of the channel-mitochondria interface 48. Similarly, in the vascular system, endothelial mechanosensors transduce disturbed shear stress into mitochondrial ROS production. This "mechano-oxidative" stress promotes endothelial inflammation and atherosclerosis, highlighting the therapeutic potential of targeting mitochondrial downstream effectors to preserve vascular integrity 49-51.

Furthermore, the interplay between mechanotransduction and immunometabolism is critical for tissue repair. Dysregulated mechanosensing in immune cells, such as macrophages, alters their mitochondrial metabolism, thereby influencing inflammation resolution and the progression of diseases like sepsis and diabetic complications 52-54. Targeting these specific mitochondrial checkpoints has shown promise; for example, bioactive compounds and targeted delivery systems that modulate this axis have been effective in alleviating vascular aging and metabolic disorders 55-57.

Conversely, physiological mechanical loading (e.g., exercise) exerts protective effects by enhancing mitochondrial quality control (MQC), a process partly mediated by myokines such as Irisin. Mechanical stimulation of skeletal muscle upregulates PGC-1α, driving the secretion of Irisin, which in turn promotes mitochondrial biogenesis and optimizes mitochondrial dynamics (fission/fusion) 58. Crucially, Irisin has been shown to enhance autophagy/mitophagy pathways, clearing damaged mitochondria to prevent the accumulation of ROS and maintain Musculoskeletal health 59. This evidence suggests that restoring the balance of the mechano-mitochondrial axis—either through exercise-mimetic myokines or pharmacological modulators of channel activity—represents a novel strategy to treat pathologies rooted in mechanotransduction failure.

### Modulatory and Multifunctional Mechanosensors

Beyond primary mechanosensors, other ion channel families contribute indirectly to mechanotransduction by modulating the muscle microenvironment, cellular excitability, and ion homeostasis.

Acid-sensing ion channels (ASICs), particularly the ASIC3 subtype, function as dual sensors in the skeletal muscle niche 60,61. While primarily recognized as proton-gated channels that detect exercise-induced acidosis (lactate/ATP), they are also proposed to sense mechanical force via a tether-based mechanism 60,62,63. This dual sensitivity allows ASIC3 to encode metabolic and mechanical stress simultaneously, mediating exercise-induced pain signals. Crucially, the loss of ASIC3 leads to exacerbated muscle injury after exhaustive exercise, suggesting a protective role in limiting tissue damage under stress 64.

The two-pore domain potassium (K2P) family functions as "background" leak channels essential for stabilizing the resting membrane potential 65. In skeletal muscle, mechanosensitive K2P members, such as TREK-1 and TRAAK, are upregulated during differentiation 66. Their physiological relevance is highlighted during myogenesis: pharmacological inhibition impairs myoblast fusion by disrupting the background K⁺ current and subsequent Ca²⁺ dynamics. By fine-tuning cellular excitability, K2P channels ensure the orderly progression of muscle development and prevent hyperexcitability-induced damage 66.

Finally, the Transmembrane (TMEM) superfamily regulates calcium handling at multiple levels, including ORAI channels and SR-localized TMEM38/TRIC channels 67. Within this superfamily, the OSCA/TMEM63 proteins have been identified as evolutionarily conserved mechanosensitive ion channels 68,69. Unlike Piezo channels which respond to minute physiological forces, biophysical studies in other tissues indicate that TMEM63 channels possess distinct "high-threshold" characteristics, requiring significant membrane tension or hyperosmotic stimuli for activation 70,71.

### Functional Coupling and Synergy Among Mechanosensitive Channels

The mechanosensitive ion channels described above do not operate as isolated entities but constitute a complex, interconnected signaling network. While they are not structurally homologous, they establish profound structural connections through physical co-localization. This spatial proximity is not coincidental but functional, facilitating rapid signal transmission and allowing local Ca²⁺ gradients to act as a physical bridge between distinct channel families.

A prime example of this coupling is the interaction between Piezo1 and TRPV4. Piezo1 serves as the primary sensor of rapid mechanical forces. Its activation triggers a transient Ca²⁺ influx, which subsequently gates the adjacent TRPV4 channels—likely through Ca²⁺-dependent phospholipase activation. This sequential Piezo1-Ca²⁺-TRPV4 axis functions as an amplification mechanism, converting brief mechanical stimuli into sustained intracellular Ca²⁺ elevation 18,72,73.

In the nervous system, proprioception relies on the synergy between Piezo2 and Acid-Sensing Ion Channels (ASICs). While Piezo2 acts as the primary transducer converting muscle stretch into electrical signals, its rapidly adapting kinetics cannot sustain firing during prolonged stretch. Evidence indicates that ASIC3 structurally and functionally complements Piezo2 by providing a slowly adapting component. This cooperation ensures that the sensory apparatus maintains tonic activity during static muscle stretch, preserving the fidelity of proprioceptive signaling 74.

Furthermore, the TRPV1 channel exhibits extensive integration with the sarcoplasmic reticulum (SR). TRPV1 functionally crosstalks with the Ryanodine Receptor 1 (RyR1) on the SR membrane, regulating Ca²⁺ release from internal stores central to muscle contraction 26. Similarly, TRPV1 synergizes with α1-adrenergic receptors to upregulate thermogenic proteins, highlighting a redundancy that ensures metabolic robustness 27.

**Figure 3:**
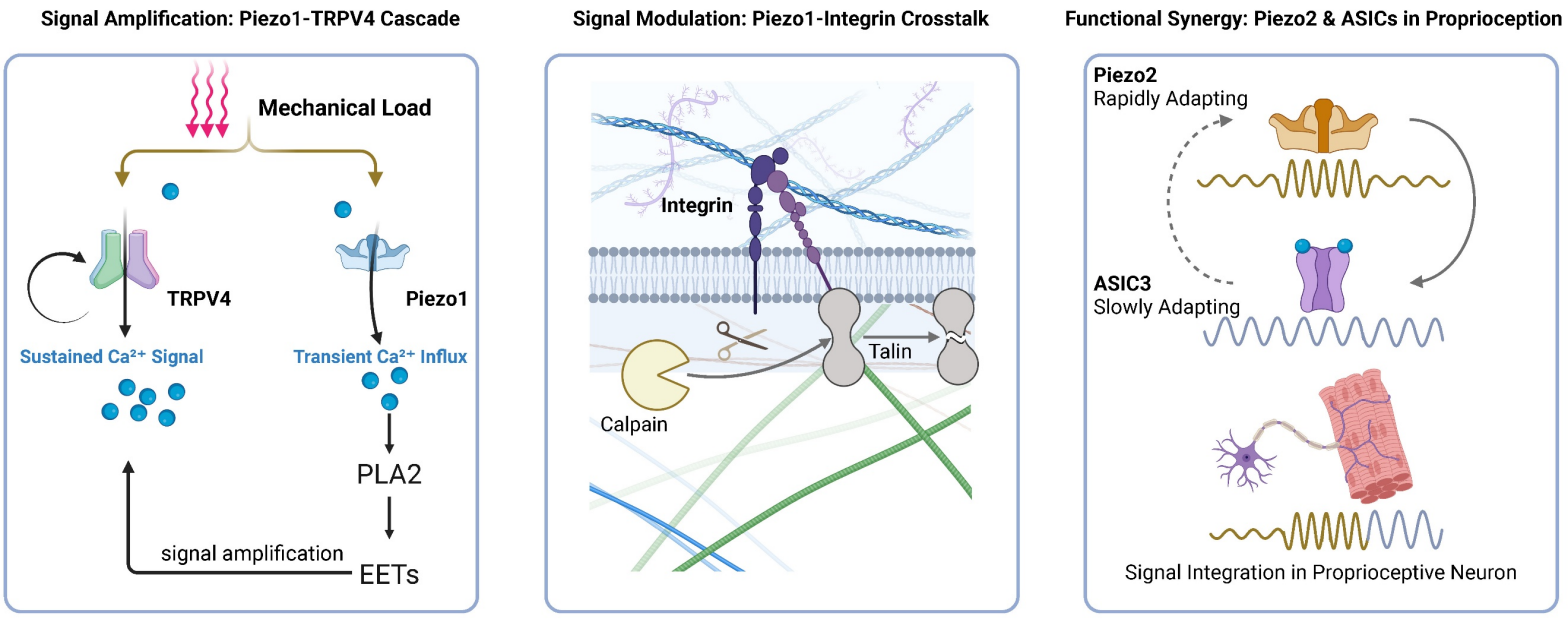
** Crosstalk and Synergy Among Mechanosensor.** (A) Signal Amplification: Mechanical force acts on both Piezo1 and TRPV4. Crucially, the Piezo1-mediated transient Ca²⁺ influx directly activates the PLA₂-EETs signaling cascade, which subsequently hyperactivates TRPV4, converting a brief mechanical stimulus into a robust, sustained Ca²⁺ signal. (B) Signal Modulation: Local Ca²⁺ influx derived from Piezo1 activates the protease calpain to cleave talin, thereby uncoupling and downregulating integrin-based focal adhesions. (C) Functional Synergy: During sustained muscle stretch, the rapidly adapting signal from Piezo2 integrates with the slowly adapting current from ASIC3—which is co-activated by mechanical tethering and local metabolites (e.g., H⁺)—to successfully encode and maintain a sustained proprioceptive firing.

In summary, mechanotransduction is orchestrated by a multi-channel network rather than a single linear pathway. From the Ca²⁺-mediated amplification between Piezo1 and TRPV4 to the temporal complementarity of Piezo2 and ASICs, these interactions form the basis of muscle plasticity. As illustrated in Figure 3, intracellular Ca²⁺ serves as the central integrator in this network, bridging primary mechanosensation with downstream effector pathways.

## Activation Mechanisms and Therapeutic Applications of Physical Stimuli

This section focuses on how distinct physical stimuli—mechanical, electrical, and thermal—selectively activate mechanosensitive ion channels in skeletal muscle, converting external energy into intracellular biological signals that drive therapeutic outcomes.

### Low-Intensity Pulsed Ultrasound (LIPUS): Precision Control via Acoustic Waves

LIPUS functions as a non-invasive mechanotherapy that transmits pulsed acoustic energy to target tissues, inducing biological effects primarily through non-thermal mechanical forces such as acoustic radiation and streaming 75. These physical cues create localized membrane deformations that are transduced into biochemical signals via two convergent pathways.

The first pathway is the Piezo1-TRPV4 signaling axis. Mechanical force directly gates Piezo1, triggering a transient Ca²⁺ influx that activates phospholipase A₂ (PLA₂). This event generates lipid second messengers (e.g., EETs) which subsequently open TRPV4 channels 72,90. This sequential relay converts the rapid, millisecond-scale opening of Piezo1 into a sustained, robust Ca²⁺ elevation mediated by TRPV4, effectively amplifying the initial mechanical stimulus to drive downstream signaling 18,91.

Parallel to this, Voltage-Gated Calcium Channels (VGCCs) act as critical signal amplifiers. As detailed in Section 2.5, the primary mechanosensor TRPC1 responds to ultrasound by generating a depolarizing current, which secondarily gates adjacent VGCCs. This "TRPC1-VGCC axis" is indispensable for specific therapeutic outcomes, including COX2 upregulation and the enhancement of mesenchymal stromal cell tropism 43.

The Ca²⁺ influx orchestrated by these pathways converges on the calcineurin/NFAT signaling hub, a master regulator of myogenic differentiation. This pathway is exemplified by the exercise-induced protein CSRP3, which utilizes calcineurin/NFAT signaling to promote hypertrophy and the slow-twitch fiber transition 92. Similarly, the circular RNA circMEF2A1 relies on this axis to drive myogenic programming 77. Thus, LIPUS achieves therapeutic precision by translating acoustic mechanics into specific Ca²⁺-dependent gene regulatory networks.

#### Electrical Stimulation: Electromechanical Coupling

Electrical stimulation is a standard therapeutic modality for preserving muscle mass during disuse or denervation 78,80,82. Its primary mechanism involves the direct depolarization of the sarcolemma, which activates L-type VGCCs 79. The resulting Ca²⁺ influx mimics physiological excitation-contraction coupling (ECC), thereby stimulating anabolic processes such as myotube maturation and protein synthesis 79,82.

Crucially, the therapeutic efficacy of electrical stimulation extends beyond voltage gating to involve a secondary mechanotransductive component. The muscle contractions induced by electrical stimulation impose a mechanical load on the fibers, which indirectly recruits mechanosensitive channels. Notably, electrical stimulation has been shown to upregulate Piezo1 expression in atrophic muscle 80. This mechanically driven Piezo1 activation engages the Akt/mTOR pathway, synergizing with VGCC-mediated signals to enhance protein synthesis and counteract atrophy 80,93. Therefore, electrical stimulation exerts a dual-modality effect: it directly triggers voltage sensors to initiate contraction, while simultaneously recruiting mechanosensors through the induced physical activity to reinforce anabolic signaling.

### Thermal Therapy: Temperature-Dependent Regulation of Ion Channels

Heat stress elicits metabolic adaptations in skeletal muscle by engaging a specific subset of thermosensitive ion channels. TRPV1 serves as the primary molecular heat sensor; its activation by therapeutic temperatures (~41°C) triggers Ca²⁺-dependent signaling that potentiates the mTOR pathway, leading to increased protein synthesis 94. Furthermore, TRPV1 activation induces PGC-1α expression, thereby enhancing mitochondrial biogenesis and respiratory capacity, effectively mimicking the metabolic benefits of exercise 95.

In contrast to excitatory channels like TRPV1, the TREK/TRAAK subfamily of K2P channels acts as cellular stabilizers in response to thermal stimuli. These background K⁺ channels are critical for maintaining the resting membrane potential in skeletal muscle cells, and they show dramatically increased activity in the physiological to mildly hyperthermic range of 37-42 °C 84. By increasing K⁺ efflux, these channels counteract depolarization and temper cellular excitability during thermal stress.

Beyond direct thermosensors, temperature profoundly modulates the gating kinetics of primary mechanosensors, creating a cross-modal regulatory landscape. Piezo1 function is significantly inhibited by cold, as lower temperatures increase membrane lipid stiffness, raising the channel's activation threshold. Conversely, Piezo2, as the principal proprioceptor, exhibits enhanced mechanosensitivity under moderate cooling 96. This dualistic response reflects their functional divergence, with Piezo1 being optimized for the stable core body temperature and Piezo2 adapted to integrate thermal and mechanical signals from the external environment 96.

### Mechanical Vibration: Frequency-Dependent Specificity

Mechanical vibration is a therapeutic strategy used to counteract muscle atrophy and weakness 97 by engaging distinct mechanosensitive pathways in a parameter-dependent manner 86. The biological outcome is highly dependent on frequency, amplitude, and duration, which determine the selective activation of specific ion channels and reflex pathways 98.

This modality operates through two distinct cellular targets. First, Piezo2 in muscle spindles entrains to vibration-induced stretch, triggering the tonic vibration reflex (TVR) which enhances neural drive and neuromuscular control 87,89. Second, Piezo1 in myofibers and satellite cells functions as a direct transducer of oscillatory stress. Studies in muscle-specific knockout models demonstrate that Piezo1 is required for the anabolic effects of vibration; its activation stimulates the Akt/mTOR and CaMKβ/AMPK pathways, thereby suppressing protein degradation and promoting regeneration 88,97,99. This specificity suggests that vibration parameters can be tuned to selectively recruit Piezo isoforms for targeted rehabilitation outcomes.

## Downstream Signaling Pathways: Intracellular Transduction and Integration of Mechanical Signals

After mechanosensitive channels convert physical stimuli into biochemical signals, the precise transmission and integration of this information depend on a series of complex downstream signaling networks 100. These networks not only decode the intensity, type, and duration of mechanical forces but also integrate multiple signals through crosstalk, ultimately coordinating key cellular decisions such as proliferation, differentiation, and survival 100. This chapter explores three core signaling axes—Hippo-YAP/TAZ, MAPK, and PI3K-Akt-mTOR/PGC-1α—elucidating how ion influx (particularly Ca²⁺) triggers these cascades to drive muscle adaptation and systemic communication.

### The Hippo-YAP/TAZ Signaling Axis: A Central Regulator of Mechanotransduction

The Hippo pathway, with its effectors YAP and TAZ, functions as a conserved sensor of tissue mechanics and organ size 63,101. Its key effectors, the transcriptional co-activators YAP and its paralog TAZ, are central molecules in sensing and integrating mechanical signals from the microenvironment 102,103. In its canonical state, a kinase cascade involving MST1/2 and LATS1/2 results in the phosphorylation of YAP and TAZ 104,105. This phosphorylation leads to their sequestration in the cytoplasm or targets them for degradation, thereby suppressing their transcriptional activity 64,104.

Mechanistically, mechanical stimuli regulate this axis primarily by modulating cytoskeletal tension. On stiff substrates or under high tension, integrin clustering activates RhoA-ROCK signaling to promote actin polymerization. This stiff cytoskeleton inhibits the upstream LATS1/2 kinases, leading to YAP/TAZ dephosphorylation and nuclear translocation. Conversely, soft environments or mechanical unloading promote LATS activity, sequestering YAP/TAZ in the cytoplasm 106-108. Crucially, this pathway is directly gated by mechanosensitive ion channels. For instance, Piezo1-mediated Ca²⁺ influx modulates actin dynamics to facilitate YAP nuclear entry 109. In skeletal muscle, this axis is pivotal for regeneration: YAP promotes the proliferation of activated satellite cells (MuSCs) post-injury, while TAZ drives their subsequent differentiation 110-113. However, chronic overactivation of YAP/TAZ in fibroblasts can drive fibrosis, highlighting the need for balanced mechanotransduction 114. This makes the YAP/TAZ-TEAD transcriptional complex a key therapeutic target for developing anti-fibrotic drugs 101,104.

### The MAPK Cascade: A Decoder of Mechanical Stress

The MAPK network translates diverse mechanical cues into specific cellular programs through its three subfamilies: ERK, JNK, and p38 111. The ERK pathway is typically activated by physiological, growth-promoting stimuli (e.g., cyclic stretch) and drives proliferation and survival via transcription factors like AP-1 111,115,116. In contrast, the JNK and p38 pathways function as stress-activated kinases, responding primarily to high-intensity or injurious forces (e.g., overstretch) to trigger inflammation or apoptosis 117. This differential activation allows muscle cells to distinguish between "training signals" (ERK-dominant) and "damage signals" (JNK/p38-dominant), a balance critical for tissue adaptation 116.

### The PI3K-Akt-mTOR Signaling Pathway: A Master Regulator of Protein Synthesis

The PI3K-Akt-mTOR pathway functions as the master switch for protein synthesis and load-induced hypertrophy 64,111,118. Mechanical loading triggers Akt activation, which phosphorylates TSC2 to relieve inhibition on mTORC1. Activated mTORC1 then phosphorylates p70S6K and 4E-BP1, driving the translation of myofibrillar proteins 111,119. Parallel to synthesis, Akt suppresses protein degradation by phosphorylating FoxO transcription factors, preventing the expression of atrogenes (Atrogin-1, MuRF1) 120.

Crucially, mechanical signaling also regulates the muscle's secretory function. Beyond hypertrophy, mechanosensitive Ca²⁺ influx (e.g., via TRPV1 or Piezo1) activates the Calcium/Calmodulin-dependent Protein Kinase (CaMK) and calcineurin pathways 121,122. These effectors upregulate Peroxisome proliferator-activated receptor-gamma coactivator-1 alpha (PGC-1α), the master regulator of mitochondrial biogenesis 95. PGC-1α not only enhances oxidative capacity but also drives the expression of FNDC5, the precursor of the myokine irisin 123. This "Mechano-Ca²⁺-PGC-1α-Irisin" axis serves as the molecular link between physical stimulation and systemic endocrine effects, explaining how localized muscle loading can trigger whole-body metabolic benefits.

## Resolution of Mechanical Signal Properties by Signaling Pathways

Signaling networks downstream of mechanoreceptors must resolve the specific properties of mechanical forces, including their type, intensity, and duration, to generate appropriate biological outputs. Signaling networks downstream of mechanoreceptors must resolve the specific properties of mechanical forces, including their type, intensity, and duration, to generate appropriate biological outputs. The type of physical force is primarily resolved by the Hippo-YAP/TAZ pathway through its sensitivity to cytoskeletal tension. Tensile forces increase cytoskeletal tension and promote actin polymerization, which inhibits upstream LATS kinases and allows YAP to enter the nucleus to drive pro-growth transcriptional programs. Conversely, compressive forces generally relieve cytoskeletal tension, thereby activating LATS kinases to sequester YAP in the cytoplasm and induce growth arrest, allowing the cell to precisely distinguish between stretch and compression 110,124-127.

The intensity of the mechanical signal is predominantly decoded by the MAPK cascade, which employs an ultrasensitivity switch mechanism to differentiate between physiological and pathological loads. Moderate mechanical forces typically activate the pro-survival ERK pathway, promoting cellular proliferation and adaptation. However, when the mechanical force exceeds a specific physiological threshold and becomes injurious, the network abruptly shifts to activate the stress-responsive JNK and p38 pathways. This threshold-dependent divergence ensures that cells mount a survival response to normal loading while initiating inflammatory or apoptotic programs only when subjected to damaging high-intensity stress 128.

Finally, the duration and temporal pattern of mechanical stimuli are integrated primarily through the PI3K-Akt-mTOR pathway, relying heavily on the dynamics of intracellular calcium signaling. A brief or transient mechanical event generates a short-lived calcium spike that may only be sufficient to trigger local cytoskeletal adjustments. In contrast, sustained or repetitive mechanical loading induces prolonged calcium elevations or oscillations. This sustained calcium influx is required to stably engage the PI3K-Akt axis and fully activate the mTORC1 complex, ensuring that massive protein synthesis and muscle hypertrophy occur only in response to persistent mechanical demand 129-131.

## Integration and Crosstalk

These pathways do not operate in isolation but form a highly interconnected network. Crosstalk occurs at multiple levels: (1) Upstream: Rho GTPases and the cytoskeleton simultaneously regulate YAP/TAZ and MAPK 132; (2) Kinase Level: The MEK/ERK pathway can activate mTORC1 independently of PI3K/Akt 118,133; and (3) Transcriptional Level: Akt-mediated phosphorylation can influence YAP stability 111,134.

**Figure 4:**
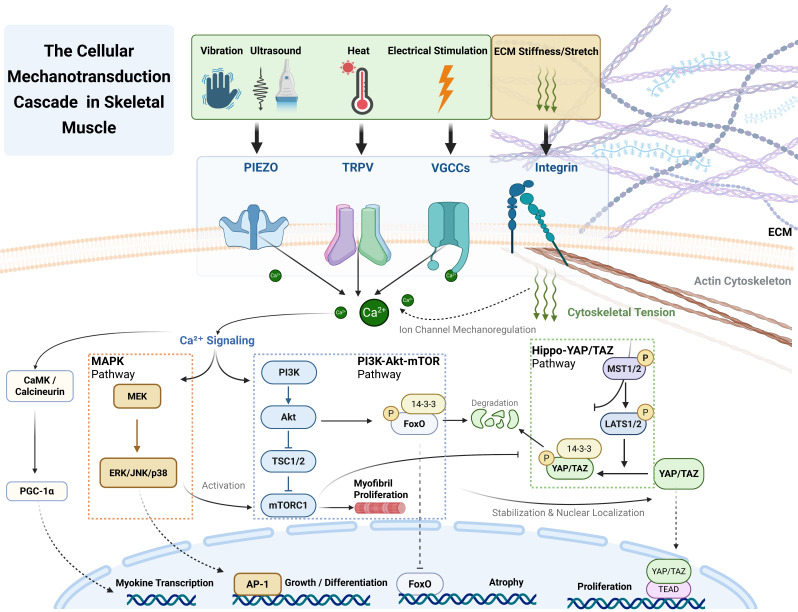
** A comprehensive model of the integrated mechanotransduction cascade.** This figure illustrates how mechanical signals flow from external physical stimuli, via a dual sensing system at the membrane (ion channels and integrins), into highly interconnected downstream networks. Beyond the core Hippo-YAP/TAZ, MAPK, and PI3K-Akt-mTOR pathways that coordinate local biological outputs (e.g., proliferation, differentiation, and hypertrophy), the model highlights the critical "mechanico-endocrine coupling" mechanism. Specifically, mechanosensitive Ca²⁺ influx activates parallel CaMK and calcineurin signaling to promote PGC-1α-mediated myokine gene transcription. Simultaneously, Ca²⁺ triggers the vesicular exocytosis of mature myokines, providing the essential molecular bridge between local mechanical loading and systemic inter-organ crosstalk.

Furthermore, as highlighted in Section 4.3, this intracellular integration extends to secretory outputs, where mechanical stress triggers the release of myokines (e.g., irisin) to communicate with distant organs. A comprehensive model of this integrated cascade—from mechanosensors to myokine secretion—is illustrated in Figure 4.

## Systemic Regulatory Roles of Skeletal Muscle: Inter-Organ Crosstalk

**Figure 5:**
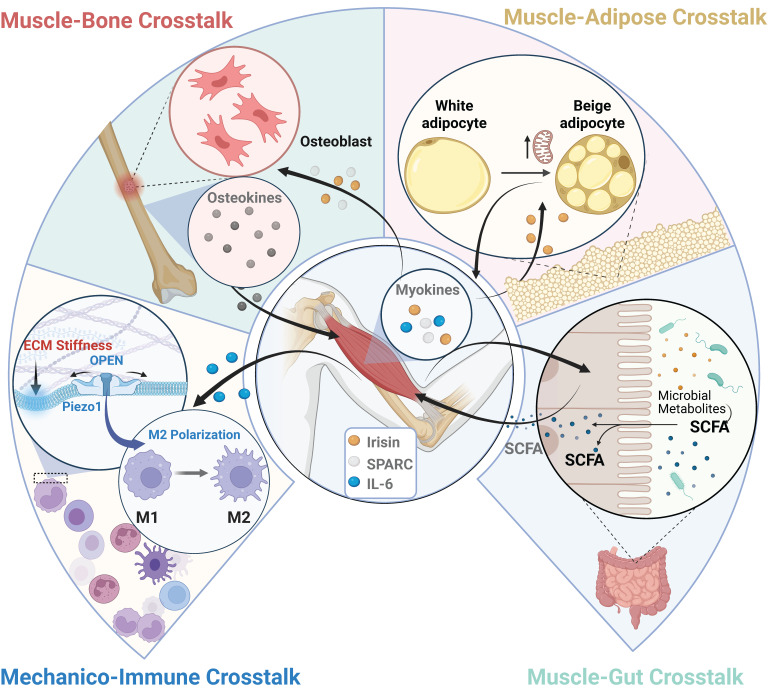
** Skeletal muscle as a mechanosensitive endocrine hub: mechanically driven inter-organ crosstalk.** Following the intracellular mechanotransduction and secretory cascades detailed in Figure 4, mechanical load stimulates skeletal muscle to release specific myokines (e.g., Irisin, SPARC, IL-6) into the circulation. These signaling molecules act on distant target organs—including bone, adipose tissue, the immune system, and the gut microbiome. In turn, these organs secrete reciprocal endocrine factors (such as osteocalcin from bone, adiponectin from adipose tissue, and short-chain fatty acids [SCFAs] from the gut) back to the muscle. This establishes a robust, bidirectional mechanico-endocrine feedback loop that orchestrates systemic metabolic and immunological homeostasis.

Beyond its fundamental role in locomotion and force generation, skeletal muscle is now recognized as a major endocrine organ 135,136. Because the synthesis and secretion of myokines are closely coupled with mechanotransduction, localized physical stimuli (e.g., exercise, ultrasound, or electrical stimulation) can trigger widespread systemic adaptations. This chapter explores how targeting muscle with mechanical energy orchestrates communication with bone, adipose, immune, and gut systems, as summarized in Figure 5.

### Skeletal Muscle as a Mechanosensitive Endocrine Organ

The foundation of muscle's systemic influence lies in its ability to function as a mechanosensitive endocrine hub, governed by a "Mechanico-Endocrine Coupling" mechanism. Mechanical stimuli activate surface mechanosensors (such as Piezo1 and VGCCs) to induce a rapid Ca²⁺ influx. This Ca²⁺ signal acts as a master switch: it promotes the gene expression of myokines via downstream pathways (e.g., p38 MAPK and PGC-1α) and directly acts on calcium-sensing proteins (e.g., Synaptotagmin) to drive the vesicular exocytosis of mature myokines 137. Through this mechanical gating, skeletal muscle releases a highly specific profile of myokines into the circulation. For instance, irisin, cleaved from the membrane protein FNDC5, is strongly induced by the mechanically activated Ca²⁺-PGC-1α signaling axis 137,138. Alongside irisin, interleukin-6 (IL-6) is transiently released during muscle contraction via calcium-dependent exocytosis, acting as an anti-inflammatory and metabolic myokine distinct from its classical immune role 139. Furthermore, mechanical loading inhibits myostatin (MSTN), a negative regulator of muscle mass; this inhibition derepresses the AMPK-PGC-1α pathway to further amplify irisin secretion 140,141. By translating physical forces into these circulating biochemical signals, skeletal muscle exerts profound regulatory effects on distant organs.

### Muscle-Bone Interactions: Bidirectional Signaling

A critical example of inter-organ communication is the extensive and bidirectional signaling that occurs between skeletal muscle and bone 135,136. This crosstalk is essential for the integrated health of the musculoskeletal system.

Mechanical activation stimulates the release of myokines that directly influence bone metabolism. For instance, myokines such as Irisin—which is synthesized and secreted following the activation of the mechanosensitive Ca²⁺-PGC-1α axis—and Secreted Protein Acidic and Rich in Cysteine (SPARC) exert anabolic effects on bone by promoting the differentiation and mineralization activity of osteoblasts 135. Conversely, bone releases osteokines, such as uncarboxylated osteocalcin, which binds to muscle receptors to promote protein synthesis and glucose uptake 135,136. Because of this intricate relationship, mechanical loading (via exercise or therapeutic physical stimuli) is a crucial regulator of the muscle-bone unit, strongly enhancing the reciprocal secretion of myokines and osteokines to counteract conditions like osteosarcopenia 135,136,142,143.

### Muscle-Adipose Crosstalk

The dialogue between skeletal muscle and adipose tissue governs key systemic metabolic processes, including lipolysis, insulin sensitivity, and thermogenesis 59,144.

Mechanically induced myokines profoundly reshape the adipose phenotype. Circulating Irisin binds to α-integrin receptors on adipocytes, activating the FAK and p38 MAPK pathways to upregulate UCP1. This induces the "browning" of white adipose tissue (WAT), transforming energy-storing cells into thermogenic, energy-expending cells 137,138,145,146. Similarly, muscle-derived IL-6 acts via the STAT3 signaling axis to stimulate lipolysis and further promote WAT browning 139,147.

In a reciprocal feedback loop, adipose tissue secretes adipokines that regulate muscle metabolism. Adiponectin enhances insulin sensitivity and stimulates fatty acid oxidation in muscle, primarily through the activation of AMPK 148-150. Leptin exerts similar metabolic benefits. However, in states of obesity, disrupted adipokine profiles (e.g., leptin resistance driven by SOCS3 overexpression) fail to properly activate muscle AMPK, fostering chronic inflammation and skeletal muscle insulin resistance 151,152.

### Muscle-Immune Interactions: Mechanico-Immune Coupling

Muscle regeneration following mechanical micro-damage depends on a precisely orchestrated immune response, particularly the M1-to-M2 polarization switch of macrophages 153,154.

Recent evidence reveals that this immune response is directly influenced by mechanical signals from the tissue microenvironment. Macrophages are inherently mechanosensitive and express channels like Piezo1, allowing them to sense physical cues such as extracellular matrix stiffness 155-157. Piezo1-mediated mechanosensing in macrophages modulates their polarization, driving a shift toward the anti-inflammatory and pro-regenerative M2 phenotype 155. Thus, the mechanical forces generated during muscle contraction directly modulate immune cell behavior to facilitate efficient repair.

### Muscle-Gut Crosstalk

Complex and bidirectional communication exists between skeletal muscle and the gut microbiome, forming an emerging frontier known as the muscle-gut axis. Regular physical exercise profoundly impacts the gut microbiota, enriching diversity and beneficial butyrate-producing species 158,159.

This influence is both endocrine and metabolic. Endocrinologically, mechanical loading triggers the release of myokines (IL-6, irisin, BDNF) that exert systemic anti-inflammatory effects and improve intestinal barrier function 59,158,160-162. Metabolically, during strenuous mechanical loading, contracting skeletal muscle releases systemic metabolites, such as lactate, into the circulation. These muscle-derived metabolites can cross the intestinal barrier and serve as direct substrates for specific gut microbiota (e.g., *Veillonella*) to synthesize short-chain fatty acids (SCFAs) like propionate 163-165.

The communication of the muscle-gut axis is bidirectional 144. In return, the gut microbiota produces a large volume of SCFAs (acetate, propionate, butyrate) that are absorbed into the bloodstream to act as signaling molecules on skeletal muscle 158. Crucially, the ability of skeletal muscle to utilize these microbial metabolites is actively regulated by mechanotransduction. Applied mechanical loading (such as exercise) activates the AMPK-PGC-1α signaling pathway, which directly upregulates the expression of Monocarboxylate Transporter 1 (MCT1) on the sarcolemma 163. This mechanically-induced upregulation of MCT1 significantly enhances the muscle's capacity to uptake and utilize gut-derived SCFAs 163. Once internalized, SCFAs affect muscle cells by activating surface G-protein-coupled receptors (e.g., GPR43) and inhibiting intracellular histone deacetylases (HDACs) 166-168. These signals converge to enhance mitochondrial biogenesis, improve energy efficiency, and increase insulin sensitivity 144,159.

## Conclusion and Future Perspectives

The ability of skeletal muscle to sense its mechanical environment is fundamental to its function and repair. This review synthesized how mechanosensitive channels—primarily Piezo, TRP, and VGCCs—act as primary molecular transducers, converting physical stimuli into electrochemical signals. These signals are integrated by downstream networks (e.g., Hippo, MAPK, PI3K-Akt-mTOR) to orchestrate local muscle adaptation and, through mechanically-gated myokine secretion, systemic inter-organ homeostasis.

Looking forward, this mechanico-endocrine framework offers profound contributions to future medical development. Translating physical therapies into targeted pharmacological interventions—such as "exercise mimetics" that modulate Piezo1 or TRPV1—could deliver the anabolic and metabolic benefits of mechanical loading to patients unable to exercise due to aging or neuromuscular disease. Furthermore, leveraging these mechanosensory pathways to selectively enhance the release of beneficial myokines (e.g., irisin) provides a novel clinical paradigm for treating systemic conditions like type 2 diabetes and osteosarcopenia.

Despite this promising potential, significant limitations and missing evidence must be addressed before clinical feasibility is achieved. A critical challenge is defining the precise "therapeutic window" for mechanotherapies; because excessive mechanotransduction is detrimental, "over-activating" channels like Piezo1 risks pathological intracellular Ca²⁺ overload and subsequent mitochondrial dysfunction. Additionally, there is a severe lack of in vivo evidence mapping channel kinetics within the 3D environment of human muscle. Finally, the ubiquitous expression of these mechanosensors poses a high risk of off-target effects, necessitating the development of muscle-specific targeted delivery systems. Cracking this "mechanical code" to safely balance anabolic signaling and excitotoxicity will be essential for developing next-generation mechanosensory medicines.

## Figures and Tables

**Table 1:** Summary of Physical Therapy Mechanisms.

Therapy	Physical Stimulus	Primary Target Channels	Key Biological Effects
Low-Intensity Pulsed Ultrasound (LIPUS)	Acoustic radiation force & streaming 75	Piezo1 (Primary sensor) 72,76 TRPV4 (Signal amplifier) 72 TRPC1/VGCCs (Secondary axis) 43	Promotes myogenic differentiation 77 Accelerates muscle repair
Electrical Stimulation	Membrane depolarization 78	VGCCs(Cav1.1, Cav1.3) 79 Piezo1 (Indirect mechanical activation) 80	Prevents/attenuates muscle atrophy 81 Promotes myofiber maturation 79 Induces fiber type shift 82
Thermal Therapy	Tissue temperature increase (~39-43°C) 83	TRPV1(Heat sensor) 83 K2P (TREK-1/TRAAK) (Mechano-thermo sensor) 84.	Inhibits atrophy pathways 85 Stabilizes membrane potential 84.
MechanicalVibration	Oscillatory motion and stress 86	Piezo2 (muscle spindles) 87 TRPV4 (Myofibers/MuSCs) 88	Attenuates disuse atrophy 86 Enhances neuromuscular function 89

## References

[1] Yaganoglu S, Kalyviotis K, Vagena-Pantoula C, Jülich D, Gaub BM, Welling M (2023). Highly specific and non-invasive imaging of Piezo1-dependent activity across scales using GenEPi. Nat Commun.

[2] Tsuchiya Y, Matsuo H, Asahara H, Inui M (2025). Matrix stiffness regulates Mkx expression in rat tenocyte through TRPM7. Biochem Biophys Rep.

[3] Tao J, Choudhury MI, Maity D, Kim T, Sun SX, Fan C-M (2023). Mechanical compression creates a quiescent muscle stem cell niche. Commun Biol.

[4] Qin Y, Yu D, Wu D, Dong J, Li WT, Ye C (2023). Cryo-EM structure of TMEM63C suggests it functions as a monomer. Nat Commun.

[5] Ponce A, Ogazon Del Toro A, Jimenez L, Roldan ML, Shoshani L (2024). Osmotically Sensitive TREK Channels in Rat Articular Chondrocytes: Expression and Functional Role. Int J Mol Sci.

[6] Qin L, He T, Chen S, Yang D, Yi W, Cao H (2021). Roles of mechanosensitive channel Piezo1/2 proteins in skeleton and other tissues. Bone Res.

[7] Du S, Liu K (2025). Mechanosensitive ion channels and inflammation: key links in cellular signal transduction. Inflamm Res.

[8] Shu H, Gao Y, Zhang Q, Sun H, Wang H, Jing C (2025). Electric currents in disc health: The role of ion channels in intervertebral disc pathophysiology. J Orthop Translat.

[9] Kefauver JM, Ward AB, Patapoutian A (2020). Discoveries in structure and physiology of mechanically activated ion channels. Nature.

[10] Chuang Y-C, Chen C-C (2022). Force From Filaments: The Role of the Cytoskeleton and Extracellular Matrix in the Gating of Mechanosensitive Channels. Front Cell Dev Biol. 2022;10. doi:10.3389/fcell.

[11] Sadoshima J, Takahashi T, Jahn L, Izumo S (1992). Roles of mechano-sensitive ion channels, cytoskeleton, and contractile activity in stretch-induced immediate-early gene expression and hypertrophy of cardiac myocytes. Proc Natl Acad Sci U S A.

[12] Nomura T, Cranfield CG, Deplazes E, Owen DM, Macmillan A, Battle AR (2012). Differential effects of lipids and lyso-lipids on the mechanosensitivity of the mechanosensitive channels MscL and MscS. Proc Natl Acad Sci U S A.

[13] Richardson J, Kotevski A, Poole K (2022). From stretch to deflection: the importance of context in the activation of mammalian, mechanically activated ion channels. The FEBS Journal.

[14] Brohawn SG (2015). How ion channels sense mechanical force: insights from mechanosensitive K2P channels TRAAK, TREK1, and TREK2. Annals of the New York Academy of Sciences.

[15] Wei F, Flowerdew K, Kinzel M, Perotti LE, Asiatico J, Omer M (2022). Changes in interstitial fluid flow, mass transport and the bone cell response in microgravity and normogravity. Bone Res.

[16] Lau P, Vico L, Rittweger J (2022). Dissociation of Bone Resorption and Formation in Spaceflight and Simulated Microgravity: Potential Role of Myokines and Osteokines?. Biomedicines.

[17] Jiang Y, Guan Y, Lan Y, Chen S, Li T, Zou S (2021). Mechanosensitive Piezo1 in Periodontal Ligament Cells Promotes Alveolar Bone Remodeling During Orthodontic Tooth Movement. Front Physiol.

[18] Jia Q, Yang Y, Chen X, Yao S, Hu Z (2022). Emerging roles of mechanosensitive ion channels in acute lung injury/acute respiratory distress syndrome. Respir Res.

[19] Shutova MS, Boehncke W-H (2022). Mechanotransduction in Skin Inflammation. Cells.

[20] Alzugaray ME, Gavazzi MV, Griffo L, Ronderos JR (2025). Piezo proteins, mechano reception and behaviour in Hydra. Sci Rep.

[21] Jagasia R, Wagner KR (2022). Piezo1: opening the way to preventing muscle atrophy. J Clin Invest.

[22] Liu Y, Cao X, Zhou Q, Deng C, Yang Y, Huang D (2024). Mechanisms and Countermeasures for Muscle Atrophy in Microgravity. Cells.

[24] Lafoux A, Lotteau S, Huchet C, Ducreux S (2020). The Contractile Phenotype of Skeletal Muscle in TRPV1 Knockout Mice is Gender-Specific and Exercise-Dependent. Life (Basel).

[25] Huang T, Chen X, He J, Zheng P, Luo Y, Wu A (2024). Eugenol mimics exercise to promote skeletal muscle fiber remodeling and myokine IL-15 expression by activating TRPV1 channel. Elife.

[26] Ding X, Zhu C, Lu Q, Zhang Y, Gao B (2025). TRPV1 signaling in skeletal muscle: A mini review of physiological and pathological roles. Cell Calcium.

[27] Abdillah AM, Lee JY, Lee YR, Yun JW (2025). Modulatory roles of capsaicin on thermogenesis in C2C12 myoblasts and the skeletal muscle of mice. Chem Biol Interact.

[28] Conte E, Romano A, De Bellis M, de Ceglia M, Rosaria Carratù M, Gaetani S (2021). Oxtr/TRPV1 expression and acclimation of skeletal muscle to cold-stress in male mice. J Endocrinol.

[29] Kurth F, Franco-Obregón A, Casarosa M, Küster SK, Wuertz-Kozak K, Dittrich PS (2015). Transient receptor potential vanilloid 2-mediated shear-stress responses in C2C12 myoblasts are regulated by serum and extracellular matrix. FASEB J.

[30] Fukazawa A, Hori A, Hotta N, Katanosaka K, Estrada JA, Ishizawa R (2023). Antagonism of TRPV4 channels partially reduces mechanotransduction in rat skeletal muscle afferents. J Physiol.

[31] Gifford JR, Ives SJ, Park S-Y, Andtbacka RHI, Hyngstrom JR, Mueller MT (2014). α1- and α2-adrenergic responsiveness in human skeletal muscle feed arteries: the role of TRPV ion channels in heat-induced sympatholysis. Am J Physiol Heart Circ Physiol.

[32] Uda Y, Azab E, Sun N, Shi C, Pajevic PD (2017). Osteocyte Mechanobiology. Curr Osteoporos Rep.

[33] Jiang Y, Zhang H, Wang J, Liu Y, Luo T, Hua H (2022). Targeting extracellular matrix stiffness and mechanotransducers to improve cancer therapy. J Hematol Oncol.

[34] Gonzalez-Valdivieso J, Ciccone G, Dhawan U, Quon T, Barcelona-Estaje E, Rodrigo-Navarro A (2025). NaBC1 Boron Transporter Enables Myoblast Response to Substrate Rigidity via Fibronectin-Binding Integrins. Adv Sci (Weinh).

[35] Boppart MD, Mahmassani ZS (2019). Integrin signaling: linking mechanical stimulation to skeletal muscle hypertrophy. Am J Physiol Cell Physiol.

[36] Yu Y, Leng Y, Song X, Mu J, Ma L, Yin L (2023). Extracellular Matrix Stiffness Regulates Microvascular Stability by Controlling Endothelial Paracrine Signaling to Determine Pericyte Fate. Arterioscler Thromb Vasc Biol.

[37] Rolvien T, Amling M (2022). Disuse Osteoporosis: Clinical and Mechanistic Insights. Calcif Tissue Int.

[38] Lei M, Wang W, Zhang H, Gong J, Wang Z, Cai H (2023). Cell-cell and cell-matrix adhesion regulated by Piezo1 is critical for stiffness-dependent DRG neuron aggregation. Cell Rep.

[39] Huang J, Pan X, Yan N (2024). Structural biology and molecular pharmacology of voltage-gated ion channels. Nat Rev Mol Cell Biol.

[40] Vergnol A, Bourguiba A, Bauché S, Traoré M, Gelin M, Gentil C (2025). Identification of CaVβ1 Isoforms Required for Neuromuscular Junction Formation and Maintenance. Cells.

[41] Garcia N, Hernández P, Lanuza MA, Tomàs M, Cilleros-Mañé V, Just-Borràs L (2022). Involvement of the Voltage-Gated Calcium Channels L- P/Q- and N-Types in Synapse Elimination During Neuromuscular Junction Development. Mol Neurobiol.

[42] Gao W, Hasan H, Anderson DE, Lee W (2022). The Role of Mechanically-Activated Ion Channels Piezo1, Piezo2, and TRPV4 in Chondrocyte Mechanotransduction and Mechano-Therapeutics for Osteoarthritis. Front Cell Dev Biol.

[43] Burks SR, Lorsung RM, Nagle ME, Tu T-W, Frank JA (2019). Focused ultrasound activates voltage-gated calcium channels through depolarizing TRPC1 sodium currents in kidney and skeletal muscle. Theranostics.

[44] Gees M, Colsoul B, Nilius B (2010). The role of transient receptor potential cation channels in Ca2+ signaling. Cold Spring Harb Perspect Biol.

[45] Machamer JB, Vazquez-Cintron EJ, Stenslik MJ, Pagarigan KT, Bradford AB, Ondeck CA (2023). Neuromuscular recovery from botulism involves multiple forms of compensatory plasticity. Front Cell Neurosci.

[46] Chang X, Zhou H, Hu J, Ge T, He K, Chen Y (2024). Targeting mitochondria by lipid-selenium conjugate drug results in malate/fumarate exhaustion and induces mitophagy-mediated necroptosis suppression. Int J Biol Sci.

[47] Wang J, Zhuang H, Li C, Cai R, Shi H, Pang B (2025). Ligustrazine nano-drug delivery system ameliorates doxorubicin-mediated myocardial injury via piezo-type mechanosensitive ion channel component 1-prohibitin 2-mediated mitochondrial quality surveillance. J Nanobiotechnology.

[48] Pu X, Liu J, Wang Y, Guan X, Wu Q, Zhang Q (2025). Ginsenoside Rb1 attenuates coronary microvascular inflammatory injury via NDUFS4-SIRT5-DUSP1-mediated mitochondrial quality control in a murine ischemia-reperfusion model. J Ginseng Res.

[49] Wang J, Zhuang H, Jia L, He X, Zheng S, Ji K (2024). Nuclear receptor subfamily 4 group A member 1 promotes myocardial ischemia/reperfusion injury through inducing mitochondrial fission factor-mediated mitochondrial fragmentation and inhibiting FUN14 domain containing 1-depedent mitophagy. Int J Biol Sci.

[50] Wang J, Pu X, Zhuang H, Guo Z, Wang M, Yang H (2025). Astragaloside IV alleviates septic myocardial injury through DUSP1-Prohibitin 2 mediated mitochondrial quality control and ER-autophagy. J Adv Res.

[51] Pang B, Dong G, Pang T, Sun X, Liu X, Nie Y (2024). Advances in pathogenesis and treatment of vascular endothelial injury-related diseases mediated by mitochondrial abnormality. Front Pharmacol.

[52] Wu Q, Wang Y, Liu J, Guan X, Chang X, Liu Z (2024). Microtubules and cardiovascular diseases: insights into pathology and therapeutic strategies. Int J Biochem Cell Biol.

[53] Chang X, Zhou S, Huang Y, Liu J, Wang Y, Guan X (2025). Zishen Huoxue decoction (ZSHX) alleviates ischemic myocardial injury (MI) via Sirt5-β-tubulin mediated synergistic mechanism of “mitophagy-unfolded protein response” and mitophagy. Chin J Nat Med.

[54] Pang B, Dong G, Pang T, Sun X, Liu X, Nie Y (2024). Emerging insights into the pathogenesis and therapeutic strategies for vascular endothelial injury-associated diseases: focus on mitochondrial dysfunction. Angiogenesis.

[55] Chang X, Zhang Q, Pu X, Liu J, Wang Y, Guan X (2024). The effect of unhealthy lifestyle on the pathogenesis of sick sinus syndrome: A life-guiding review. Medicine (Baltimore).

[56] Pu X, Wu Q, Yan Z, Zhou S, Zhang Q, Zhang X (2025). Tanshinone IIA modulates Sirt5 and Metll3 interaction to govern mitochondria-endoplasmic reticulum unfolded protein response in coronary microvascular injury. Phytomedicine.

[57] Chang X, Zhou S, Yan Z, Zhang Q, Liu J, Wang Y (2025). Potential Candidates of Natural Antioxidants From Herbs for Treating Lung Disorders: Focus on Redox Balance and Natural Products. Phytother Res.

[58] Yang K, Zhang P, Li J, Zhang G, Chang X (2024). Potential of natural drug modulation of endoplasmic reticulum stress in the treatment of myocardial injury. J Pharm Anal.

[59] Zhao C, Wu Y, Zhu S, Liu H, Xu S (2024). Irisin Protects Musculoskeletal Homeostasis via a Mitochondrial Quality Control Mechanism. Int J Mol Sci.

[60] Cheng Y-R, Jiang B-Y, Chen C-C (2018). Acid-sensing ion channels: dual function proteins for chemo-sensing and mechano-sensing. J Biomed Sci.

[61] Khataei T, Benson CJ (2023). ASIC3 plays a protective role in delayed-onset muscle soreness (DOMS) through muscle acid sensation during exercise. Front Pain Res (Lausanne).

[62] Cristofori-Armstrong B, Budusan E, Rash LD (2021). Mambalgin-3 potentiates human acid-sensing ion channel 1b under mild to moderate acidosis: Implications as an analgesic lead. Proc Natl Acad Sci U S A.

[63] Wu Y, Wang S, Guo Z, Sun M, Xu Z, Du Y (2024). Hapalindole Q suppresses autophagosome-lysosome fusion by promoting YAP1 degradation via chaperon-mediated autophagy. Proc Natl Acad Sci U S A.

[64] Heng BC, Zhang X, Aubel D, Bai Y, Li X, Wei Y (2020). Role of YAP/TAZ in Cell Lineage Fate Determination and Related Signaling Pathways. Front Cell Dev Biol.

[65] Bustos D, Bedoya M, Ramírez D, Concha G, Zúñiga L, Decher N (2020). Elucidating the Structural Basis of the Intracellular pH Sensing Mechanism of TASK-2 K2P Channels. Int J Mol Sci.

[66] Afzali AM, Ruck T, Herrmann AM, Iking J, Sommer C, Kleinschnitz C (2016). The potassium channels TASK2 and TREK1 regulate functional differentiation of murine skeletal muscle cells. Am J Physiol Cell Physiol.

[67] Zhang N, Pan H, Liang X, Xie J, Han W (2022). The roles of transmembrane family proteins in the regulation of store-operated Ca2+ entry. Cell Mol Life Sci.

[68] Zhou X, Park KH, Yamazaki D, Lin P-H, Nishi M, Ma Z (2020). TRIC-A Channel Maintains Store Calcium Handling by Interacting With Type 2 Ryanodine Receptor in Cardiac Muscle. Circ Res.

[69] Shan Y, Zhang M, Chen M, Guo X, Li Y, Zhang M (2024). Activation mechanisms of dimeric mechanosensitive OSCA/TMEM63 channels. Nat Commun.

[70] Zhang M, Shan Y, Cox CD, Pei D (2023). A mechanical-coupling mechanism in OSCA/TMEM63 channel mechanosensitivity. Nat Commun.

[71] Murthy SE, Dubin AE, Whitwam T, Jojoa-Cruz S, Cahalan SM, Mousavi SAR (2018). OSCA/TMEM63 are an Evolutionarily Conserved Family of Mechanically Activated Ion Channels. Elife.

[72] Swain SM, Liddle RA (2023). Mechanosensing Piezo channels in gastrointestinal disorders. J Clin Invest.

[73] Endesh N, Chuntharpursat-Bon E, Revill C, Yuldasheva NY, Futers TS, Parsonage G (2023). Independent endothelial functions of PIEZO1 and TRPV4 in hepatic portal vein and predominance of PIEZO1 in mechanical and osmotic stress. Liver Int.

[74] Woo S-H, Lukacs V, de Nooij JC, Zaytseva D, Criddle CR, Francisco A (2015). Piezo2 is the principal mechanotransduction channel for proprioception. Nat Neurosci.

[75] Uddin SMZ, Qin Y-X (2013). Enhancement of osteogenic differentiation and proliferation in human mesenchymal stem cells by a modified low intensity ultrasound stimulation under simulated microgravity. PLoS One.

[76] Guan M, Zhang X, Li X, Liao B, Han W, Tan J (2025). Research Progress of Osteoarthritis Treatment by Low Intensity Pulsed Ultrasound. Smart Med.

[77] Shen X, Zhao X, He H, Zhao J, Wei Y, Chen Y (2023). Evolutionary conserved circular MEF2A RNAs regulate myogenic differentiation and skeletal muscle development. PLoS Genet.

[78] Long Y, Li J, Yang F, Wang J, Wang X (2021). Wearable and Implantable Electroceuticals for Therapeutic Electrostimulations. Adv Sci (Weinh).

[79] Yang GH, Kim W, Kim J, Kim G (2021). A skeleton muscle model using GelMA-based cell-aligned bioink processed with an electric-field assisted 3D/4D bioprinting. Theranostics.

[80] Jia W, Wang T, Chen F, Liu Z, Hou X, Cao W (2025). Low-Intensity Pulsed Ultrasound Responsive Scaffold Promotes Intramembranous and Endochondral Ossification via Ultrasonic, Thermal, and Electrical Stimulation. ACS Nano.

[81] Pan L-L, Ke J-Q, Zhao C-C, Huang S-Y, Shen J, Jiang X-X (2016). Electrical Stimulation Improves Rat Muscle Dysfunction Caused by Chronic Intermittent Hypoxia-Hypercapnia via Regulation of miRNA-Related Signaling Pathways. PLoS One.

[82] Zhou X, Li G, Wu D, Liang H, Zhang W, Zeng L (2023). Recent advances of cellular stimulation with triboelectric nanogenerators. Exploration (Beijing).

[83] Hwang S-M, Jo Y-Y, Cohen CF, Kim Y-H, Berta T, Park C-K (2022). Venom Peptide Toxins Targeting the Outer Pore Region of Transient Receptor Potential Vanilloid 1 in Pain: Implications for Analgesic Drug Development. Int J Mol Sci.

[84] Rueda-Ruzafa L, Herrera-Pérez S, Campos-Ríos A, Lamas JA (2021). Are TREK Channels Temperature Sensors?. Front Cell Neurosci.

[85] Ogura Y, Naito H, Tsurukawa T, Ichinoseki-Sekine N, Saga N, Sugiura T (2007). Microwave hyperthermia treatment increases heat shock proteins in human skeletal muscle. Br J Sports Med.

[86] Wang P, Yang X, Yang Y, Yang L, Zhou Y, Liu C (2015). Effects of whole body vibration on pain, stiffness and physical functions in patients with knee osteoarthritis: a systematic review and meta-analysis. Clin Rehabil.

[87] Chwała W, Pogwizd P, Rydzik Ł, Ambroży T (2021). Effect of Vibration Massage and Passive Rest on Recovery of Muscle Strength after Short-Term Exercise. Int J Environ Res Public Health.

[88] Mirzoev TM (2023). The emerging role of Piezo1 channels in skeletal muscle physiology. Biophys Rev.

[89] Paolucci T, Agostini F, Bernetti A, Paoloni M, Mangone M, Santilli V (2021). Integration of focal vibration and intra-articular oxygen-ozone therapy in rehabilitation of painful knee osteoarthritis. J Int Med Res.

[90] Phan MN, Leddy HA, Votta BJ, Kumar S, Levy DS, Lipshutz DB (2009). Functional characterization of TRPV4 as an osmotically sensitive ion channel in porcine articular chondrocytes. Arthritis Rheum.

[91] Pyrshev K, Atamanchuk-Stavniichuk A, Kordysh M, Zaika O, Tomilin VN, Pochynyuk O (2024). Independent regulation of Piezo1 activity by principal and intercalated cells of the collecting duct. J Biol Chem.

[92] Mutryn MF, Brannick EM, Fu W, Lee WR, Abasht B (2015). Characterization of a novel chicken muscle disorder through differential gene expression and pathway analysis using RNA-sequencing. BMC Genomics.

[93] Lin C-Y, Sassi A, Wu Y, Seaman K, Tang W, Song X (2025). Mechanotransduction pathways regulating YAP nuclear translocation under Yoda1 and vibration in osteocytes. Bone.

[94] Lin C-Y, Song X, Ke Y, Raha A, Wu Y, Wasi M (2022). Yoda1 Enhanced Low-Magnitude High-Frequency Vibration on Osteocytes in Regulation of MDA-MB-231 Breast Cancer Cell Migration. Cancers (Basel).

[95] Milici A, Talavera K (2021). TRP Channels as Cellular Targets of Particulate Matter. Int J Mol Sci.

[96] Zheng W, Nikolaev YA, Gracheva EO, Bagriantsev SN (2019). Piezo2 integrates mechanical and thermal cues in vertebrate mechanoreceptors. Proc Natl Acad Sci U S A.

[97] Du X, Gamper N (2013). Potassium channels in peripheral pain pathways: expression, function and therapeutic potential. Curr Neuropharmacol.

[98] Delafontaine A, Vialleron T, Fischer M, Laffaye G, Chèze L, Artico R (2019). Acute Effects of Whole-Body Vibration on the Postural Organization of Gait Initiation in Young Adults and Elderly: A Randomized Sham Intervention Study. Front Neurol.

[99] Nakazawa Y, Petrova RS, Sugiyama Y, Nagai N, Tamura H, Donaldson PJ (2021). Regulation of the Membrane Trafficking of the Mechanosensitive Ion Channels TRPV1 and TRPV4 by Zonular Tension, Osmotic Stress and Activators in the Mouse Lens. Int J Mol Sci.

[100] Montagner M, Dupont S (2020). Mechanical Forces as Determinants of Disseminated Metastatic Cell Fate. Cells.

[101] Hu L, Sun Y, Liu S, Erb H, Singh A, Mao J (2022). Discovery of a new class of reversible TEA domain transcription factor inhibitors with a novel binding mode. Elife.

[102] Guo X, Zhao B (2013). Integration of mechanical and chemical signals by YAP and TAZ transcription coactivators. Cell Biosci.

[103] Ji J, Xu R, Zhang X, Han M, Xu Y, Wei Y (2018). Actin like-6A promotes glioma progression through stabilization of transcriptional regulators YAP/TAZ. Cell Death Dis.

[104] Papavassiliou KA, Sofianidi AA, Papavassiliou AG (2024). YAP/TAZ-TEAD signalling axis: A new therapeutic target in malignant pleural mesothelioma. J Cell Mol Med.

[105] Zhang X, Zhao H, Li Y, Xia D, Yang L, Ma Y (2018). The role of YAP/TAZ activity in cancer metabolic reprogramming. Mol Cancer.

[106] Yu F-X, Guan K-L (2013). The Hippo pathway: regulators and regulations. Genes Dev.

[107] Lecoutre S, Lambert M, Drygalski K, Dugail I, Maqdasy S, Hautefeuille M (2022). Importance of the Microenvironment and Mechanosensing in Adipose Tissue Biology. Cells.

[108] Scott LE, Weinberg SH, Lemmon CA (2019). Mechanochemical Signaling of the Extracellular Matrix in Epithelial-Mesenchymal Transition. Front Cell Dev Biol.

[109] Ozkan AD, Wijerathne TD, Gettas T, Lacroix JJ (2023). Force-induced motions of the PIEZO1 blade probed with fluorimetry. Cell Rep.

[110] Hansen CG, Moroishi T, Guan K-L (2015). YAP and TAZ: a nexus for Hippo signaling and beyond. Trends Cell Biol.

[111] Fischer M, Rikeit P, Knaus P, Coirault C (2016). YAP-Mediated Mechanotransduction in Skeletal Muscle. Front Physiol.

[112] Schaub C, Rose M, Frasch M (2019). Yorkie and JNK revert syncytial muscles into myoblasts during Org-1-dependent lineage reprogramming. J Cell Biol.

[113] Han J, Zhang J, Zhang X, Luo W, Liu L, Zhu Y (2024). Emerging role and function of Hippo-YAP/TAZ signaling pathway in musculoskeletal disorders. Stem Cell Res Ther.

[114] Croft AS, Roth Y, Oswald KAC, Ćorluka S, Bermudez-Lekerika P, Gantenbein B (2021). In Situ Cell Signalling of the Hippo-YAP/TAZ Pathway in Reaction to Complex Dynamic Loading in an Intervertebral Disc Organ Culture. Int J Mol Sci.

[115] Whitmarsh AJ, Yang SH, Su MS, Sharrocks AD, Davis RJ (1997). Role of p38 and JNK mitogen-activated protein kinases in the activation of ternary complex factors. Mol Cell Biol.

[116] Lewthwaite JC, Bastow ER, Lamb KJ, Blenis J, Wheeler-Jones CPD, Pitsillides AA (2006). A Specific Mechanomodulatory Role for p38 MAPK in Embryonic Joint Articular Surface Cell MEK-ERK Pathway Regulation*. Journal of Biological Chemistry.

[117] Song F, Wang Y, Jiang D, Wang T, Zhang Y, Ma H (2016). Cyclic Compressive Stress Regulates Apoptosis in Rat Osteoblasts: Involvement of PI3K/Akt and JNK MAPK Signaling Pathways. PLoS One.

[118] Sun EJ, Wankell M, Palamuthusingam P, McFarlane C, Hebbard L (2021). Targeting the PI3K/Akt/mTOR Pathway in Hepatocellular Carcinoma. Biomedicines.

[119] Feng L, Chen Z, Bian H (2024). Skeletal muscle: molecular structure, myogenesis, biological functions, and diseases. MedComm (2020).

[120] Rao VK, Das D, Taneja R (2022). Cancer Cachexia: Signaling and Transcriptional Regulation of Muscle Catabolic Genes. Cancers (Basel).

[121] Wang Y, Zhang Z, Yang Q, Cao Y, Dong Y, Bi Y (2022). Immunoregulatory Role of the Mechanosensitive Ion Channel Piezo1 in Inflammation and Cancer. Molecules.

[122] Wang W, Huang M, Huang X, Ma K, Luo M, Yang N (2025). GsMTx4-blocked PIEZO1 channel promotes myogenic differentiation and alleviates myofiber damage in Duchenne muscular dystrophy. Skelet Muscle.

[123] Wrann CD, White JP, Salogiannnis J, Laznik-Bogoslavski D, Wu J, Ma D (2013). Exercise induces hippocampal BDNF through a PGC-1α/FNDC5 pathway. Cell Metab.

[124] Wang Y, Cao W, Cui J, Yu Y, Zhao Y, Shi J (2018). Arterial Wall Stress Induces Phenotypic Switching of Arterial Smooth Muscle Cells in Vascular Remodeling by Activating the YAP/TAZ Signaling Pathway. Cell Physiol Biochem.

[125] Rausch V, Hansen CG (2020). The Hippo Pathway, YAP/TAZ, and the Plasma Membrane. Trends Cell Biol.

[126] Plouffe SW, Lin KC, Moore JL, Tan FE, Ma S, Ye Z (2018). The Hippo pathway effector proteins YAP and TAZ have both distinct and overlapping functions in the cell. J Biol Chem.

[127] Ferraiuolo M, Verduci L, Blandino G, Strano S (2017). Mutant p53 Protein and the Hippo Transducers YAP and TAZ: A Critical Oncogenic Node in Human Cancers. Int J Mol Sci.

[128] Bleedorn JA, Hornberger TA, Goodman CA, Hao Z, Sample SJ, Amene E (2018). Temporal mechanically-induced signaling events in bone and dorsal root ganglion neurons after in vivo bone loading. PLoS One.

[129] Tripathi KP, Piccirillo M, Guarracino MR (2018). An integrated approach to infer cross-talks between intracellular protein transport and signaling pathways. BMC Bioinformatics.

[130] Sarbassov DD, Guertin DA, Ali SM, Sabatini DM (2005). Phosphorylation and regulation of Akt/PKB by the rictor-mTOR complex. Science.

[131] Hornberger TA, Stuppard R, Conley KE, Fedele MJ, Fiorotto ML, Chin ER (2004). Mechanical stimuli regulate rapamycin-sensitive signalling by a phosphoinositide 3-kinase-, protein kinase B- and growth factor-independent mechanism. Biochem J.

[132] Meli VS, Veerasubramanian PK, Downing TL, Wang W, Liu WF (2023). Mechanosensation to inflammation: Roles for YAP/TAZ in innate immune cells. Sci Signal.

[133] Miyazaki M, McCarthy JJ, Fedele MJ, Esser KA (2011). Early activation of mTORC1 signalling in response to mechanical overload is independent of phosphoinositide 3-kinase/Akt signalling. J Physiol.

[134] Shao D, Zhai P, Del Re DP, Sciarretta S, Yabuta N, Nojima H (2014). A functional interaction between Hippo-YAP signalling and FoxO1 mediates the oxidative stress response. Nat Commun.

[135] Wei Z, Ge F, Che Y, Wu S, Dong X, Song D (2021). Metabolomics Coupled with Pathway Analysis Provides Insights into Sarco-Osteoporosis Metabolic Alterations and Estrogen Therapeutic Effects in Mice. Biomolecules.

[136] Oliveri C, Xourafa A, Morabito N, Di Giovanni A, Lupo E, Basile G (2025). Calf circumference predicts changes of bone mineral density in postmenopausal osteoporotic women receiving denosumab. Aging Clin Exp Res.

[137] Zhang Y, Li R, Meng Y, Li S, Donelan W, Zhao Y (2014). Irisin stimulates browning of white adipocytes through mitogen-activated protein kinase p38 MAP kinase and ERK MAP kinase signaling. Diabetes.

[138] Zhang Y, Xie C, Wang H, Foss RM, Clare M, George EV (2016). Irisin exerts dual effects on browning and adipogenesis of human white adipocytes. Am J Physiol Endocrinol Metab.

[139] Radványi Á, Röszer T (2024). Interleukin-6: An Under-Appreciated Inducer of Thermogenic Adipocyte Differentiation. Int J Mol Sci.

[140] Knights AJ, Wu J, Tseng Y-H (2020). The Heating Microenvironment: Intercellular Cross Talk Within Thermogenic Adipose Tissue. Diabetes.

[141] Shan T, Liang X, Bi P, Kuang S (2013). Myostatin knockout drives browning of white adipose tissue through activating the AMPK-PGC1α-Fndc5 pathway in muscle. FASEB J.

[142] Leuchtmann AB, Adak V, Dilbaz S, Handschin C (2021). The Role of the Skeletal Muscle Secretome in Mediating Endurance and Resistance Training Adaptations. Front Physiol.

[143] Zhou B-N, Zhang Q, Lin X-Y, Hu J, Zhao D-C, Jiang Y (2022). The roles of sclerostin and irisin on bone and muscle of orchiectomized rats. BMC Musculoskelet Disord.

[144] Ambroszkiewicz J, Gajewska J, Mazur J, Klemarczyk W, Rowicka G, Ołtarzewski M (2021). Does a Vegetarian Diet Affect the Levels of Myokine and Adipokine in Prepubertal Children?. J Clin Med.

[145] Vliora M, Grillo E, Corsini M, Ravelli C, Nintou E, Karligiotou E (2022). Irisin regulates thermogenesis and lipolysis in 3T3-L1 adipocytes. Biochim Biophys Acta Gen Subj.

[146] Giron M, Thomas M, Dardevet D, Chassard C, Savary-Auzeloux I (2022). Gut microbes and muscle function: can probiotics make our muscles stronger?. J Cachexia Sarcopenia Muscle.

[147] Gandhi AY, Yu J, Gupta A, Guo T, Iyengar P, Infante RE (2022). Cytokine-Mediated STAT3 Transcription Supports ATGL/CGI-58-Dependent Adipocyte Lipolysis in Cancer Cachexia. Front Oncol.

[148] Maissan P, Mooij EJ, Barberis M (2021). Sirtuins-Mediated System-Level Regulation of Mammalian Tissues at the Interface between Metabolism and Cell Cycle: A Systematic Review. Biology (Basel).

[149] Yamauchi T, Kamon J, Minokoshi Y, Ito Y, Waki H, Uchida S (2002). Adiponectin stimulates glucose utilization and fatty-acid oxidation by activating AMP-activated protein kinase. Nat Med.

[150] Kraegen EW, Bruce C, Hegarty BD, Ye J-M, Turner N, Cooney G (2009). AMP-activated protein kinase and muscle insulin resistance. Front Biosci (Landmark Ed).

[151] Smith U, Kahn BB (2016). Adipose tissue regulates insulin sensitivity: role of adipogenesis, de novo lipogenesis and novel lipids. J Intern Med.

[152] Yang Z, Hulver M, McMillan RP, Cai L, Kershaw EE, Yu L (2012). Regulation of insulin and leptin signaling by muscle suppressor of cytokine signaling 3 (SOCS3). PLoS One.

[153] Shi R, Liu F, Qin Q, Li P, Huo Z, Zhou Y (2025). Bioinspired piezoelectric patch design for sonodynamic therapy: a preclinical mechanistic evaluation of rotator cuff repair and functional regeneration. Front Bioeng Biotechnol.

[154] Lee U, Stuelsatz P, Karaz S, McKellar DW, Russeil J, Deak M (2022). A Tead1-Apelin axis directs paracrine communication from myogenic to endothelial cells in skeletal muscle. iScience.

[155] Gu D, Xia Y, Ding Z, Qian J, Gu X, Bai H (2024). Inflammation in the Peripheral Nervous System after Injury. Biomedicines.

[156] Jin F, Li T, Wei Z, Xiong R, Qian L, Ma J (2022). Biofeedback electrostimulation for bionic and long-lasting neural modulation. Nat Commun.

[157] Jiang Q, Li Z, Dang D, Wei J, Wu H (2024). Role of mechanosensitive channel Piezo1 protein in intestinal inflammation regulation: A potential target. FASEB J.

[158] Cutuli D, Decandia D, Giacovazzo G, Coccurello R (2023). Physical Exercise as Disease-Modifying Alternative against Alzheimer's Disease: A Gut-Muscle-Brain Partnership. Int J Mol Sci.

[159] Altajar S, Baffy G (2020). Skeletal Muscle Dysfunction in the Development and Progression of Nonalcoholic Fatty Liver Disease. J Clin Transl Hepatol.

[160] Souza PB de, de Araujo Borba L, Castro de Jesus L, Valverde AP, Gil-Mohapel J, Rodrigues ALS (2023). Major Depressive Disorder and Gut Microbiota: Role of Physical Exercise. Int J Mol Sci.

[161] Valder S, Brinkmann C (2022). Exercise for the Diabetic Gut-Potential Health Effects and Underlying Mechanisms. Nutrients.

[162] Bi J, Zhang J, Ren Y, Du Z, Li T, Wang T (2020). Irisin reverses intestinal epithelial barrier dysfunction during intestinal injury via binding to the integrin αVβ5 receptor. J Cell Mol Med.

[163] Zhang L, Xin C, Wang S, Zhuo S, Zhu J, Li Z (2024). Lactate transported by MCT1 plays an active role in promoting mitochondrial biogenesis and enhancing TCA flux in skeletal muscle. Sci Adv.

[164] Bonen A, Tonouchi M, Miskovic D, Heddle C, Heikkila JJ, Halestrap AP (2000). Isoform-specific regulation of the lactate transporters MCT1 and MCT4 by contractile activity. Am J Physiol Endocrinol Metab.

[165] McCullagh KJ, Poole RC, Halestrap AP, O'Brien M, Bonen A (1996). Role of the lactate transporter (MCT1) in skeletal muscles. Am J Physiol.

[166] Lange O, Proczko-Stepaniak M, Mika A (2023). Short-Chain Fatty Acids-A Product of the Microbiome and Its Participation in Two-Way Communication on the Microbiome-Host Mammal Line. Curr Obes Rep.

[167] Otten BMJ, Sthijns MMJPE, Troost FJ (2023). A Combination of Acetate, Propionate, and Butyrate Increases Glucose Uptake in C2C12 Myotubes. Nutrients.

[168] Walls J, Sinclair L, Finlay D (2016). Nutrient sensing, signal transduction and immune responses. Semin Immunol.

